# Is it possible to recruit HIV self-test users for an anonymous phone-based survey using passive recruitment without financial incentives? Lessons learned from a pilot study in Côte d’Ivoire

**DOI:** 10.1186/s40814-021-00965-2

**Published:** 2022-01-06

**Authors:** Arlette Simo Fotso, Arsène Kouassi Kra, Mathieu Maheu-Giroux, Sokhna Boye, Marc d’Elbée, Odette Ky-zerbo, Nicolas Rouveau, Noel Kouassi N’Guessan, Olivier Geoffroy, Anthony Vautier, Joseph Larmarange, for the ATLAS Team

**Affiliations:** 1 Centre Population et Développement, Institut de Recherche pour le Développement, Université Paris Descartes, Inserm ERL 1244, Paris, France; 2 Department of Epidemiology and Occupational Health, School of Population and Global Health, McGill University, Montréal, QC H3A 1G1 Canada; 3 Department of Global Health and Development, Faculty of Public Health and Policy, London School of Hygiene and Tropical Medicine, London, UK; 4 Institut de Recherche pour le Développement, Transvihmi (UMI 233 IRD, 1175 INSERM, Montpellier University), Montpellier, France; 5 Solidarité Thérapeutique et Initiatives pour la Santé, Solthis, Abidjan, Côte d’Ivoire; 6 SolthisSolidarité Thérapeutique et Initiatives pour la Santé, Solthis, Dakar, Sénégal

**Keywords:** HIV/AIDS, HIV self-testing, ATLAS project, Key populations, Men who have sex with men, Female sex workers, Drug users, Secondary distribution, Monitoring, Telephone survey

## Abstract

**Background:**

Due to the discreet and private nature of HIV self-testing (HIVST), it is particularly challenging to monitor and assess the impacts of this testing strategy. To overcome this challenge, we conducted a study in Côte d’Ivoire to characterize the profile of end users of HIVST kits distributed through the ATLAS project (*AutoTest VIH, Libre d’Accéder à la connaissance de son Statut*). Feasibility was assessed using a pilot phone-based survey.

**Methods:**

The ATLAS project aims to distribute 221300 HIVST kits in Côte d’Ivoire from 2019 to 2021 through both primary (e.g., direct distribution to primary users) and secondary distribution (e.g., for partner testing). The pilot survey used a passive recruitment strategy—whereby participants voluntarily called a toll-free survey phone number—to enrol participants. The survey was promoted through a sticker on the HIVST instruction leaflet and hotline invitations and informal promotion by HIVST kit-dispensing agents. Importantly, participation was not financially incentivized, even though surveys focussed on key populations usually use incentives in this context.

**Results:**

After a 7-month period in which 25,000 HIVST kits were distributed, only 42 questionnaires were completed. Nevertheless, the survey collected data from users receiving HIVST kits via both primary and secondary distribution (69% and 31%, respectively).

**Conclusion:**

This paper provides guidance on how to improve the design of future surveys of this type. It discusses the need to financial incentivize participation, to reorganize the questionnaire, the importance of better informing and training stakeholders involved in the distribution of HIVST, and the use of flyers to increase the enrolment of users reached through secondary distribution.

## Key messages regarding feasibility

- Recruiting HIV self-test users for an anonymous phone-based survey is challenging, and it is unclear if passive recruitment without financial incentives is a viable method.
- After a 7-month period, and with 25000 kits distributed, only 42 questionnaires were completed.
- The study highlights the need for to financially incentivize participation as well as the importance of better training of stakeholders involved in the distribution of HIVST and the use of flyers to increase survey participation.

## Background

In West Africa, only 68% of people living with HIV were aware of their HIV status in 2019 [1], which is far from the 95% UNAIDS 2030 target. Knowing one’s HIV status is essential to engagement in the HIV status neutral prevention continuum. It is also a first and necessary step to enter the HIV treatment and care cascade. By receiving treatment and achieving viral load suppression, people living with HIV (PLHIV) considerably reduce their risk of morbidity and mortality and their risk of onward transmission of the virus [2].

In its efforts to help countries increase HIV testing uptake, the World Health Organization (WHO) developed global guidelines on HIV self-testing (HIVST) in 2016, recommending that this strategy to be offered as an additional approach to traditional HIV testing services [3]. The recommendation was further reinforced in the WHO’s 2019 consolidated guidelines [4]. After the successful implementation of HIVST in Eastern and Southern Africa through the *HIV Self-Testing Africa Initiative – Research* (STAR) project [5], Unitaid funded the ATLAS project (*AutoTest VIH, Libre d’Accéder à la connaissance de son Statut*) to promote self-testing and distributed approximately 400,000 HIVST kits from 2019 to 2021 in three countries in West Africa (Côte d’Ivoire, Mali, and Senegal) [6].

This region of Africa has a unique epidemiologic context, characterized by a comparatively low but still generalized prevalence of HIV in the general adult population (between 0.4% and 3%) and a high prevalence among key populations (female sex workers, men having sex with men, and people who use drugs, among others). The stigmatization experienced by these groups limits their access to traditional facility-based testing strategies [7]. Community-based outreach testing activities have been successfully implemented in West Africa but still face difficulties in reaching all members of these key populations. Considering this specific context, the ATLAS project designed a strategy that combines both primary and secondary distribution of HIVST kits. Primary distribution is a strategy whereby HIVST kits are given directly to final users for their own use, while secondary distribution refers to HIVST kits being provided to primary contacts who redistribute them to their sexual partners, peers, or clients.

**Fig. 1:**
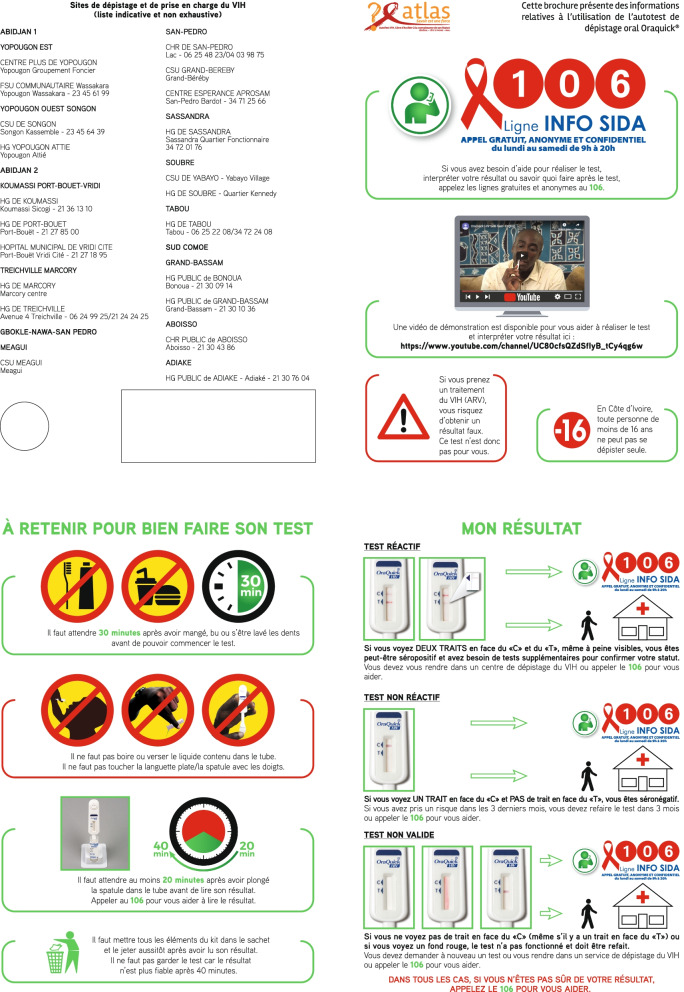
Additional instruction leaflet include in HIV self-testing (HIVST) kits distributed by the ATLAS project (2019–2020) in Côte d’Ivoire

ATLAS is implemented in partnership with the ministries of health of the three countries and national implementers already involved in HIV testing activities. Eight delivery channels have been identified to prioritize different populations (female sex workers—FSWs, men having sex with men—MSM, people who use drugs—PWUD, people living with HIV, and patients consulting for a sexually transmitted infection) and their networks, and they include both facility-based and outreach strategies [6]. In all three countries, dedicated information leaflets have been developed using cognitive interviews [8]. These leaflets (Fig. 1) are systematically distributed with all HIVST kits. They provide visual information adapted to the local context, including a link to a demonstration video, and promote the national toll-free HIV hotline (with the phone number 106 in Côte d’Ivoire), where users can obtain information and support to learn about HIVST. At the bottom left of the fourth page of the leaflet, there are coloured, numbered circular stickers to identify the distribution channel and the implementing partner for monitoring purposes.

Although it is crucial from a monitoring and evaluation point of view to document the socio-behavioural profiles and experiences of HIVST users, the private nature of HIVST makes such documentation challenging [3]. To ensure privacy and confidentiality for final users, ATLAS does not systematically track the use of HIVST kits. Dispensing agents can, and are invited to, provide their contact information to those wanting additional support to perform HIVST or interpret their HIVST results. A free national hotline is also available in all ATLAS countries. However, HIVST users have no obligation to report whether they actually used the HIVST kit or to report their test results.

To circumvent such issues, we designed an anonymous phone-based survey (named the *Coupons Survey*) that relied on ‘passive’ participant recruitment—with HIVST users invited to voluntarily call a survey toll free telephone number. The survey was designed to not interfere with HIVST kit distribution and to smoothly operate within the ATLAS project while maintaining voluntary participation, anonymity, privacy, and autonomy.

Due to the many challenges of implementing such a survey, we started with a pilot phase in Côte d’Ivoire. This paper aims to report on the lessons learned from this pilot and on the recommendations for the full implementation phase.

## Methods

The *Coupons* pilot survey used convenience sampling and was conducted in Côte d’Ivoire between November 25, 2019, and June 29, 2020. Participants were enrolled through two main recruitment streams and invited to call a toll-free survey hotline (86010), which was different from the national HIV hotline (106).

**Fig. 2:**
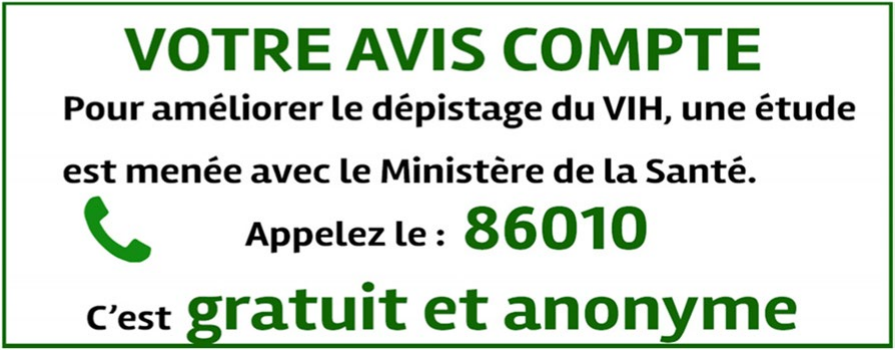
Sticker used to promote the Coupons pilot survey as part of the ATLAS project

First, during the survey period, a sticker (or ‘*coupon*’) was systematically pasted on all the ATLAS instruction leaflets (Fig. 1) near the coloured distribution channel sticker. The sticker (Fig. 2) measured 9 cm by 3.5 cm, corresponding to the available space on the HIVST instruction leaflets. Given the limited physical dimensions of the sticker, it had the following message: “Your opinion matters. To improve HIV testing, a study is being conducted with the Ministry of Health. Call 86010. It is free and anonymous” (see Fig. 2). Additionally, dispensing agents in the field were trained to promote the survey to users.

The second recruitment stream was performed through the national HIV information line (106). When a caller reported having used an HIVST kit, counsellors were trained to invite them to call the *Coupons* phone line to participate in the survey.

When individuals called the survey hotline, trained operators explained the survey, checked their eligibility, and collected verbal consent before proceeding with the interviews. The participants’ responses were recorded digitally on computers. The pilot survey collected information on all participants’ socio-demographic characteristics, sexual behaviours, experience with HIVST, and HIVST results. To be eligible for the survey, the participants were required to be of legal age to self-test for HIV in Côte d’Ivoire (16 years or older) and have previously used an HIVST kit.

The pilot survey did not include financial incentives. The relevance of incentives in surveys has been discussed widely in the literature, and there is no consensus in terms of cost-effectiveness, response quality, sample composition, response bias, or participants’ increased expectations for future surveys [9]. For these reasons, following discussion with the ATLAS Technical Advisory Group, Unitaid, and WHO, it was decided to test the feasibility of the *Coupons* survey without financial incentives in this pilot survey.

During the pilot survey period, ATLAS distributed approximately 25,000 HIVST kits in the 11 implementing health districts of Côte d’Ivoire. The target sample of the pilot survey was 2000 individuals, requiring a minimum participation rate of 8%. As per stated in the approved protocol, the sample size was set according to pragmatic and budgetary criteria [10]. The budget allowed to recruit up to 2000 individuals for this pilot phase.

## Results

At the end of the 7-month pilot period, 51 individuals had called the survey line. Among them, 42 (82%) completed the questionnaire, 3 made an appointment to answer the questions but were never reached afterward, 3 terminated the call, 2 refused to proceed, and 1 interview was interrupted due to technical issues. Of those who completed the questionnaire, 10 (24%) were referred by the hotline [11].

**Fig. 3:**
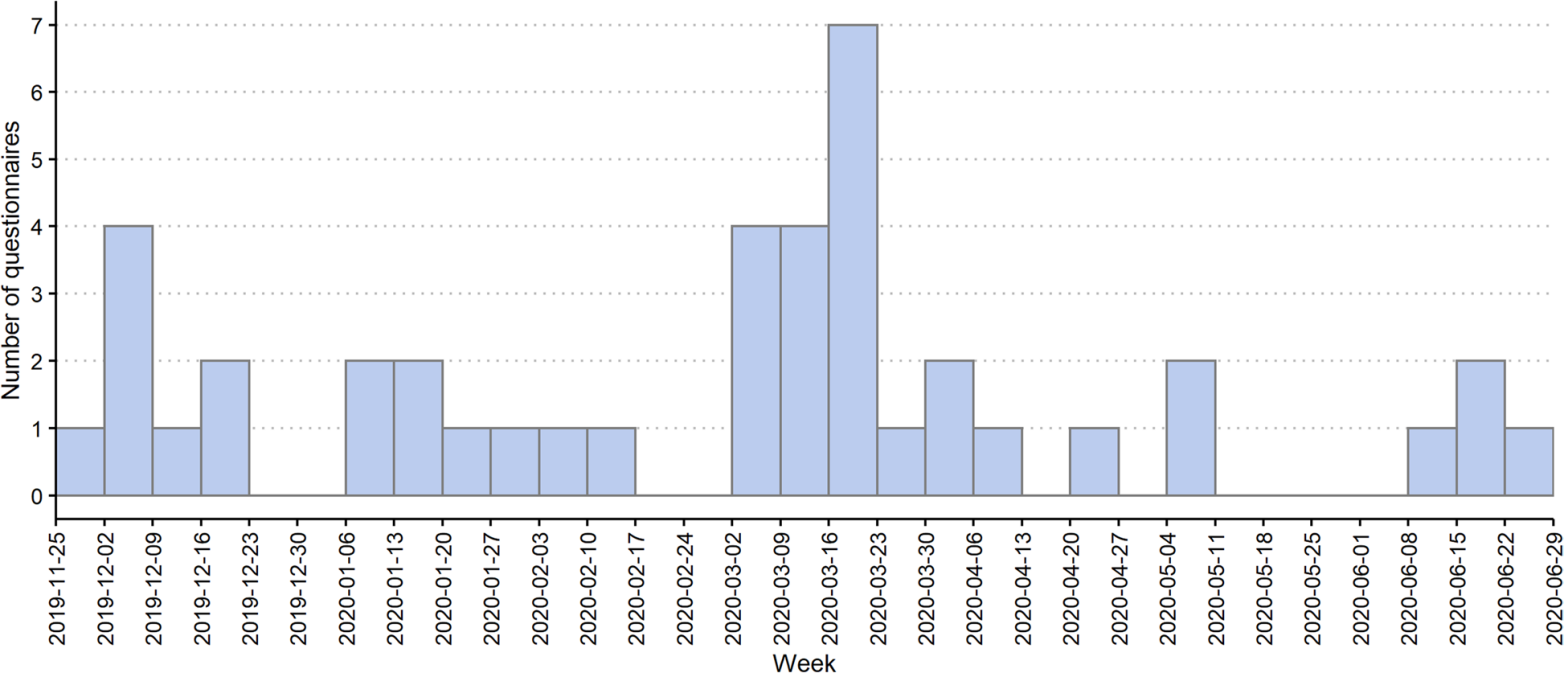
Completed questionnaires among users of HIV-self tests per week in Côte d’Ivoire as part of the ATLAS project (2019–2020)

Figure 3 shows the weekly number of participants over time. Relatively few questionnaires were completed in the first few weeks. Technical issues occurred when the line was first opened, and some participants were mistakenly charged for their phone calls. This was due to a disagreement between the two major telephone operators in Côte d’Ivoire, leading them to charge calls to each other’s tool-free numbers. The annual break for the ATLAS teams in charge of dispensing HIVST kits and the company in charge of the survey hotline occurred between the end of December 2019 and the beginning of January 2020, so no questionnaires were completed during these weeks. Afterward, survey participation remained very low, with a maximum of 2 questionnaires completed per week.

In mid-February 2020, a consortium meeting was held with all ATLAS implementing partners. Based on their feedback, it was decided to reinforce the training of dispensing agents through the routine supervision of the ATLAS team and a memory aid brochure. This measure resulted in a modest increase in the recruitment of participants.

However, at the end of March 2020, a few days after COVID-19 was declared to be a global pandemic, survey participation decreased considerably and remained relatively low until the end of the pilot study. The COVID-19 pandemic forced the operational team to adapt their activities by entirely interrupting or reducing outreach activities, which drastically reduced the number of HIVST kits distributed and, concomitantly, severely limited the number sticker invitations in circulation.

Although not representative of all those who received HIVST kits, the results of the completed questionnaires show that enrolling secondary HIVST users is feasible: 13 (31%) of interviewees received their HIVST kits through secondary distribution. The participants were evenly distributed by gender, and they were young, averaging 25 years of age.

Most participants (27 [64%]) did not report the colour or the number code indicating the channel through which they received their HIVST kits: most participants disregarded the information leaflet after having conducted the HIVST. For those for which this information was known, most received their HIVST kits through FSW-based and MSM-based outreach—6 (14%) and 4 (10%), respectively. This finding provides some evidence that ATLAS was able to reach key populations and vulnerable groups. Most of the participants—39 (93%)—reported having no troubles understanding how to perform HIVST.

## Discussion

Participation in the pilot survey was unsatisfactory due to several factors. First, the invitation sticker was small and inconspicuous, and confusion between the survey number and the hotline number might have occurred. Given the limited amount of information provided on the sticker, it is also possible that users did not understand that it was advertising a survey. The latter point is reinforced by the fact that we received feedback that people did not understand why they were being asked personal questions such as those related to their socio-demographic characteristics. The second factor is related to the mobilization of dispensing agents, particularly peer educators. Such mobilization is crucial, but it was insufficient during the pilot survey. In fact, our feedback confirms that the relevance and importance of the survey were not always perceived accurately by the dispensing agents. Third, mistrust of the survey was reinforced by the technical problems encountered at the start of the survey (problem with free access to the survey line) and the unavailability of the line for 2 weeks (annual closure). Once negative rumours circulate about an investigation, they are difficult to reverse. It is therefore essential to improve communication around the survey (using a dedicated flyer), to further mobilize dispensing agents (through dedicated training) and to allow participation through another process than by calling the hotline for those who are afraid of being charged (for example, by allowing individuals to page a number or to send a message by SMS to be called back).

Above all, the lack of financial incentives is an essential point that was raised by local partners. A majority of past surveys conducted with key populations have included financial incentives [12]. Such incentives are now expected [9] by members of key populations who are the focus of the survey. Given the very low participation in the absence of financial incentives, they seem essential to introduce going forward.

Another limitation of the pilot phase was the difficulty identifying the dispensing channel using the coloured sticker; thus, it is necessary to plan other strategies to collect this information. In addition, given the unease of the participants when asked about their socio-demographic characteristics and sexual behaviours, there is a need to review the formulation of the questions and reorganize the questionnaire in such a way that HIVST-related questions are asked first.

However, there are also some positive points. First, the survey recruited both primary and secondary contacts, which shows that its design allowed the collection of information about final users who received HIVST kits through secondary distribution. This information is particularly challenging to obtain, as it is not collected in routine programmatic data. Second, data on some primary contacts’ demographic characteristics, such as age or region, are consistent with the distribution figures reported in programmatic data, showing the potential of the survey to collect reliable data. Finally, few people were recruited via the national HIV hotline, consistent with the fact that calls to the national hotline are limited and consistent with the pilot study results (admittedly from a very small sample) indicating that HIVST users encounter few difficulties in using the test. The leaflet and the demonstration video are therefore often sufficient to obtain the necessary information. This finding is in line with the feedback from the field agents. Hence, it makes sense to abandon recruitment via the HIV hotline for the at-scale survey.

## Conclusion

In the 7-month period during which we piloted an innovative phone-based survey to characterize HIVST users, we recruited few participants: only 42 questionnaires were completed—far from our target of 2000 participants.

We derived several lessons from this feasibility pilot. First, the pilot highlights the importance of investing in the training of dispensing agents to advertise and advocate for survey participation. Second, it shows the importance of introducing financial incentives to encourage the participation of HIVST users. Third, it stresses the need to reorganize the questionnaire to introduce questions perceived as sensitive later. Fourth, it underlines the importance of publicizing the survey using dedicated materials, separate from the instruction leaflet. This strategy would provide enough space to provide the required survey details to primary and secondary users and improve the recording of information about the distribution channel.

Despite its limitations, the pilot survey allowed us to anonymously recruit secondary distribution participants who, by definition, cannot be tracked using programmatic data.

Using the lessons learned from this pilot survey, we amended the study protocol and redesigned the at-scale *Coupons* survey which was conducted between March and June 2021 in the three ATLAS countries. The amended protocol includes, among other things, (i) provision of a financial incentive of XOF 2000 per participants (West African CFA franc), (ii) training and provision of information poster to HIVST dispensing agents regarding the objectives and design of the survey, (iii) provision of flyers accompanying the HIVST kits to invite users to participate in the survey—including a unique number to prevent multiple participation by a single user and identify the distribution channel, and (iv) reorganization of the survey questionnaire [13].

## Data Availability

The dataset is available on Zenodo: https://zenodo.org/record/4687979

## Abbreviations

ATLAS: AutoTest VIH Libre d’Accéder à la connaissance de son Statut
FSWs: Female sex workers
HIVST: HIV self-testing
MSM: Men who have sex with men
PWUD: People who use drugs
STAR: HIV Self-Testing Africa Initiative – Research
STI: Sexually transmitted infection patient
XOF: West African CFA franc
